# BSG Isoform 2 (ENST00000353555) Is a Prognostic Biomarker in Predicting Unfavorable Overall Survival of Patients With Liver Hepatocellular Carcinoma

**DOI:** 10.7759/cureus.61844

**Published:** 2024-06-06

**Authors:** Wei Xiong, Ying Deng

**Affiliations:** 1 Department of Hepatobiliary Surgery, Sichuan Provincial People's Hospital, University of Electronic Science and Technology of China, Chengdu, CHN; 2 Cancer Center, Sichuan Provincial People's Hospital, University of Electronic Science and Technology of China, Chengdu, CHN

**Keywords:** overall survival, liver cancer, enst00000353555, alternative transcription, bsg

## Abstract

Background: CD147, encoded by the BSGgene, has complex transcripts that encode proteins of different lengths. Total BSG transcription is a prognostic biomarker for patients with liver cancer. This study tried to analyze the expression profile and prognostic significance of BSG transcripts in liver cancer.

Materials and methods: RNA sequencing data from The Cancer Genome Atlas (TCGA) and the Genotype-Tissue Expression (GTEx) project, survival data from TCGA, and protein expression data from the Human Protein Atlas were systematically analyzed.

Results: Among the four protein-coding transcripts of BSG*,* ENST00000353555 encoding basigin-2 is the dominant transcript isoform. It might be an independent prognostic biomarker for unfavorable overall survival in patients with liver cancer (HR: 1.404, 95% CI: 1.1224-1.754, p = 0.003).

Conclusions: ENST00000353555 might be a prognostic biomarker linking unfavorable overall survival in liver cancer patients.

## Introduction

CD147 (basigin or EMMPRIN) is a transmembrane glycoprotein encoded by the BSG gene. BSG is overexpressed in various cancers, including liver cancer. Higher BSG expression correlates with advanced tumor stages, worse differentiation, and increased metastasis and recurrence in hepatocellular carcinoma [1,2]. High BSG expression is also associated with poor prognosis in hepatocellular carcinoma patients [1,3]. Patients with higher BSG levels tend to have shorter overall survival and a higher risk of tumor recurrence after surgery [1,3]. Alternative splicing and alternative promoter usage are critical post-transcriptional regulatory mechanisms that significantly contribute to transcriptomic and proteomic diversity in human cells. Over 95% of transcribed human genes are estimated to undergo alternative splicing, leading to structural transcript variation and proteome diversity [4,5].

Previous studies have found that BSG has different transcripts encoding proteins of varying lengths [6-8]. Based on the National Center for Biotechnology Information (NCBI) data, three isoforms of basigin proteins can be transcribed by four mRNA isoforms, including NM_001728.4/ENST00000333511.9 that encodes basigin-1, NM_198589.3/ENST00000353555.9 that encodes basigin-2, and NM_198590.3/ENST00000545507.6 and NM_198591.4/ENST00000346916.9 that encode basigin-3. These isoforms exert distinct regulatory effects in cancers. In hepatocellular carcinoma, the oncogenic effects might be associated with the overexpression of isoform 2 (basigin-2) [9]. Overexpression of basigin-3 can inhibit the proliferation and invasion of hepatocellular carcinoma [10].

Based on previous findings, we hypothesized that different transcripts of BSG might exert distinct prognostic value for patients with hepatocellular carcinoma. To validate this hypothesis, this study tried to explore the expression profile and prognostic significance of BSG transcripts in liver cancer using data from The Cancer Genome Atlas (TCGA).

## Materials and methods

Immunohistochemistry data collection and image retrieval

The protein expression of basigin BSG in normal liver and liver cancer tissues was analyzed through immunohistochemistry (IHC) staining. Images for the analysis were retrieved from the Human Protein Atlas (HPA) database [4], following their requirement for citation to images. The specific URLs for the images utilized in this study are "https://images.proteinatlas.org/2427/6189_A_7_4.jpg" and "https://images.proteinatlas.org/2427/6188_B_7_8.jpg".

Data extraction

The pan-cancer dataset, which included data from TCGA and the Genotype-Tissue Expression (GTEx), was obtained from the UCSC Xena [11] data browser (https://xenabrowser.net/). Gene expression and transcript-specific expression transformed in the form of log2(TPM+0.001) were extracted and compared. Prognosis data, including overall survival (OS) and progression-free interval (PFI), were extracted for survival analysis. A total of 363 primary liver cases with OS data and 364 primary liver cases with PFI data were included for analysis.

Survival analysis

Kaplan-Meier survival curves were generated by separating the patients into two groups (median gene or transcription expression) using the "survfit" function of the R software (R Foundation for Statistical Computing, Vienna, Austria) package "survival". The Cox proportional hazards regression model [12] was generated using the package survival package (version 3.5-7) in the R software to analyze the relationship between gene or transcript expression (as a continuous variable) and prognosis (OS and PFI). The hazard ratio (HR) and 95% confidence interval (CI) were calculated.

Statistical analysis

The difference in survival curves was evaluated using the log-rank test. Univariate and multivariate Cox regression analyses were conducted to assess the independent prognostic value of ENST00000353555. One-way ANOVA followed by Tukey-Kramer post-hoc multiple comparison tests was performed to check the statistical differences among the three groups. P < 0.05 was considered statistically significant.

## Results

ENST00000353555 encoding basigin-2 is the dominant transcript isoform in liver cancer

**Figure 1:**
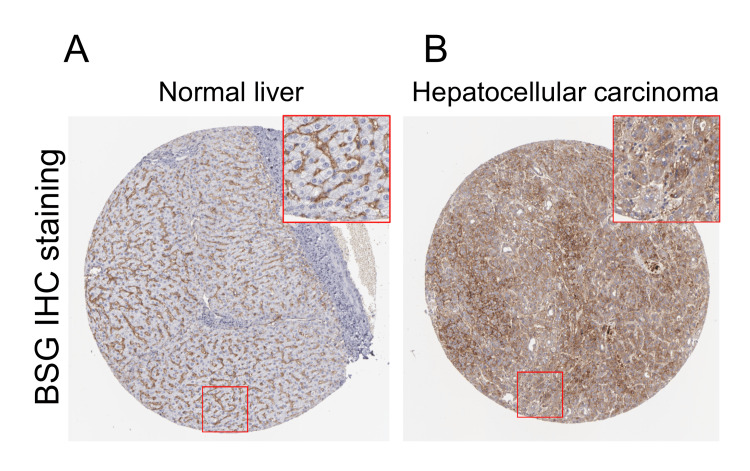
The protein expression of BSG in normal liver and liver cancer tissues. (A-B) Immunohistochemistry (IHC) staining of BSG in normal liver (A) and liver cancer tissues (B). Images were retrieved from the Human Protein Atlas (HPA) database [4]. Images available from: "https://images.proteinatlas.org/2427/6189_A_7_4.jpg" and "https://images.proteinatlas.org/2427/6188_B_7_8.jpg". Image credit: Human Protein Atlas.

**Figure 2:**
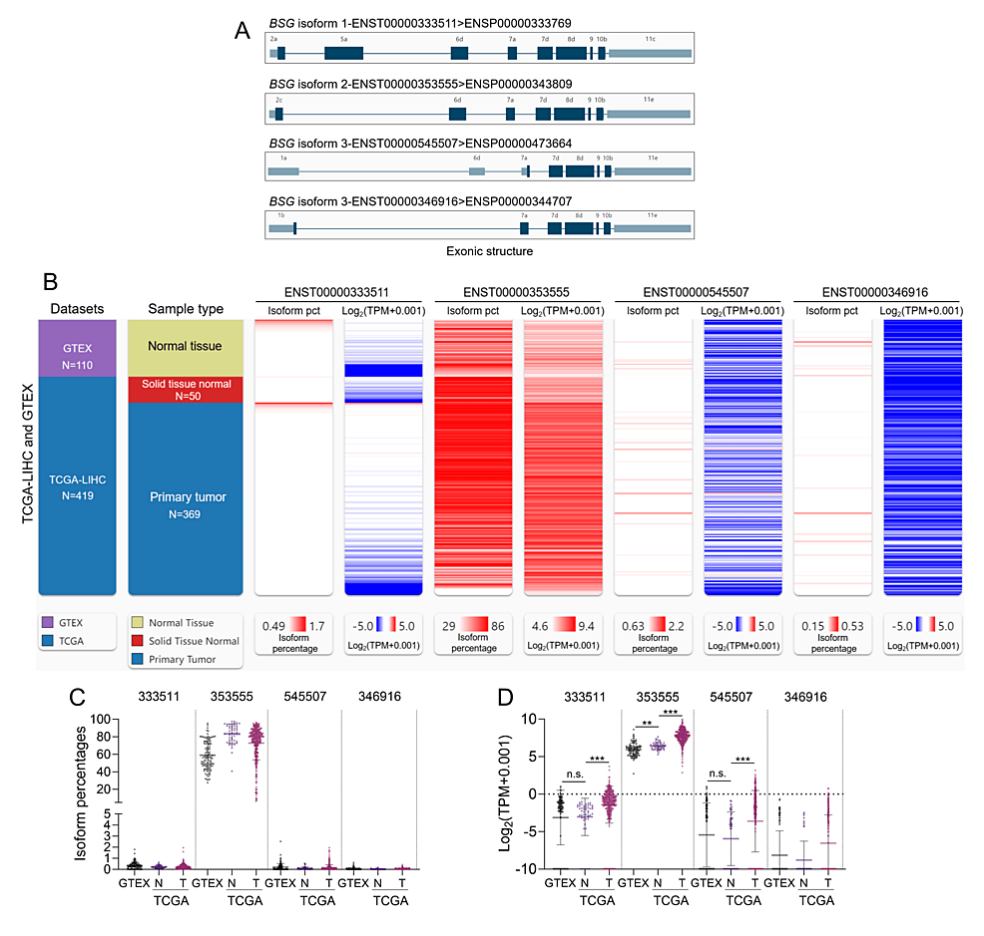
The expression profile of BSG transcripts at the mRNA levels in liver cancer. (A) The exonic structures of ENST00000333511, ENST00000353555, ENST00000545507, and ENST00000346916. (B) A heatmap showing the isoform percentage and expression of ENST00000333511, ENST00000353555, ENST00000545507, and ENST00000346916 in normal liver tissues in GTEx, and tumor-adjacent normal liver tissues and primary liver tumor tissues in TCGA. (C-D) Comparison of transcript isoform expression percentages (C) and expression (D) of ENST00000333511, ENST00000353555, ENST00000545507, and ENST00000346916 in normal liver tissues in GTEx, and tumor-adjacent normal liver tissues (N) and primary liver tumor tissues (T) in TCGA. n.s.: not significant; **: p < 0.01; ***: p < 0.001; GTEx: Genotype-Tissue Expression; TCGA: The Cancer Genome Atlas; TCGA-LIHC: The Cancer Genome Atlas Liver Hepatocellular Carcinoma.

Based on IHC staining data of BSG in the HPA, we confirmed upregulated BSG expression at the protein level in liver cancer tissues (Figure 1B) compared to normal liver tissues (Figure 1A). Then, based on gene transcript expression data in The Cancer Genome Atlas Liver Hepatocellular Carcinoma (TCGA-LIHC) and GTEx, we compared the expression of the four protein-coding transcripts (ENST00000333511, ENST00000353555, ENST00000545507, and ENST00000346916; Figure 2A) of the BSG gene in normal liver (N = 110), tumor-adjacent normal tissues (N = 50), and primary liver cancer tissues (N = 369). Both isoform expression percentages and expression levels were compared between the three groups (Figures 2B-2D). Results showed ENST00000353555 is the dominant isoform in normal, tumor-adjacent normal, and tumor tissues (Figures 2B, 2C). Nearly all cases in the tumor group had strong ENST00000353555 expression (log2(TPM+0.001)>3) (Figure 2D). Some cases in the tumor group had a moderate expression of ENST00000333511 and ENST00000545507 expression (log2(TPM+0.001)>0) (Figure 2D). The expression of these three transcripts was significantly higher in the tumor group than in the adjacent normal group (Figure 2D).

ENST00000353555 expression is an independent prognostic biomarker of unfavorable overall survival in patients with liver cancer

**Figure 3:**
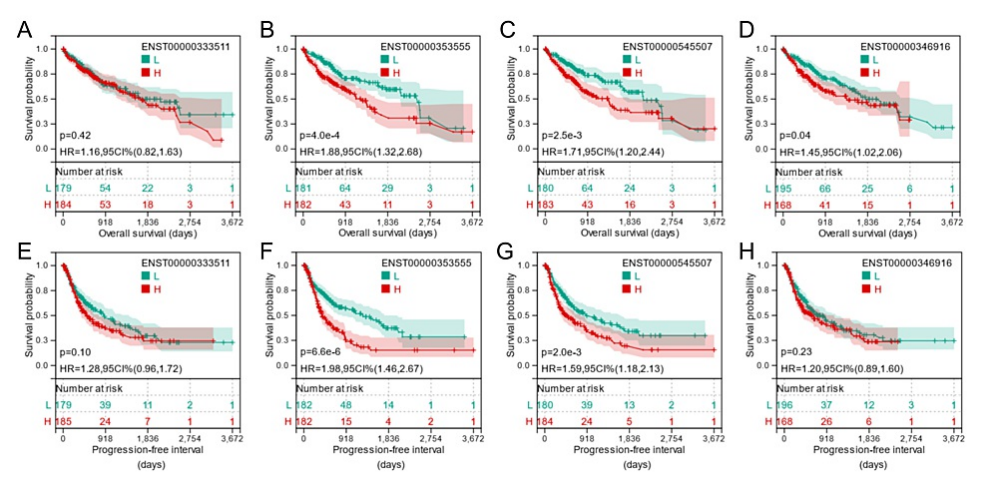
ENST00000353555 expression is an independent prognostic biomarker of unfavorable overall survival in patients with liver cancer. (A-H) Kaplan-Meier survival curves for OS (A-D) and PFI (E-H) comparisons were generated. Patients with primary liver cancer in TCGA-LIHC were divided into two groups based on the median gene or transcript expression (50%). Log-rank test was performed to analyze the statistical significance between the higher and lower expression groups. OS: overall survival; PFI: progression-free interval; HR: hazard ratio; CI: confidence interval; TCGA-LIHC: The Cancer Genome Atlas Liver Hepatocellular Carcinoma.

Kaplan-Meier survival curves for OS (Figures 3A-3D) and PFI (Figures 3E-3H) comparisons were generated to assess the survival differences in the patients with primary liver cancer, stratified by median BSG or transcript expression. Log-rank test showed a significant (p < 0.05) difference in OS and PFI by median ENST00000353555 and ENST00000545507 separation (Figures 3B, 3C, 3F, 3G). In univariate analysis, ENST00000353555 expression was associated with poor OS and PFI in patients with liver cancer (Tables 1, 2). Multivariate Cox regression analysis confirmed the independent prognostic significance of ENST00000353555 in terms of OS, after adjustment of the American Joint Committee on Cancer (AJCC) stages and residual tumors (HR: 1.404, 95%CI: 1.1224-1.754, p = 0.003) (Table 1). However, the ENST00000353555 expression had no independent prognostic value in terms of PFI (Table 2).

**Table 1:** Univariate and multivariate analysis of OS in TCGA-LIHC. OS: overall survival; TCGA-LIHC: The Cancer Genome Atlas Liver Hepatocellular Carcinoma; AJCC: American Joint Committee on Cancer.

Characteristics	Total (N)	Univariate analysis		Multivariate analysis	
		Hazard ratio (95% CI)	P-value	Hazard ratio (95% CI)	P-value
ENST00000353555	363	1.478 (1.196-1.828)	<0.001	1.404 (1.124-1.754)	0.003
Gender	363	-	-	-	-
Male	245	Reference	-	-	-
Female	118	1.213 (0.849-1.733)	0.290	-	-
Age	363	1.012 (0.998-1.026)	0.094	1.015 (0.999-1.031)	0.059
AJCC stages	339	-	-	-	-
III/IV	87	Reference	-	Reference	-
I/II	252	0.397 (0.273-0.577)	<0.001	0.408 (0.276-0.603)	<0.001
Residual tumor	356	-	-	-	-
R0	318	Reference	-	Reference	-
R1	17	1.449 (0.705-2.976)	0.313	0.628 (0.225-1.749)	0.373
RX	20	3.182 (1.528-6.626)	0.002	2.927 (1.391-6.162)	0.005
R2	1	10.824 (1.476-79.356)	0.019	17.736 (2.164-145.337)	0.007
Histological grade	358	-	-	-	-
G1	55	Reference	-	-	-
G3	117	1.205 (0.693-2.096)	0.508	-	-
G2	175	1.179 (0.696-1.998)	0.540	-	-
G4	11	1.439 (0.485-4.272)	0.512	-	-

**Table 2:** Univariate and multivariate analysis of PFI in TCGA-LIHC. PFI: progression-free interval; TCGA-LIHC: The Cancer Genome Atlas Liver Hepatocellular Carcinoma; AJCC: American Joint Committee on Cancer.

Characteristics	Total (N)	Univariate analysis		Multivariate analysis	
		Hazard ratio (95% CI)	P-value	Hazard ratio (95% CI)	P-value
ENST00000353555	364	1.226 (1.029-1.461)	0.023	1.133 (0.950-1.353)	0.166
Gender	364	-	-	-	-
Male	245	Reference	-	-	-
Female	119	1.035 (0.759-1.413)	0.827	-	-
Age	364	0.996 (0.985-1.007)	0.453	-	-
AJCC stages	340	-	-	-	-
III/IV	88	Reference	-	Reference	-
I/II	252	0.447 (0.322-0.620)	<0.001	0.461 (0.332-0.642)	<0.001
Residual tumor	357	-	-	-	-
R0	318	Reference	-	-	-
R1	17	1.577 (0.875-2.843)	0.130	-	-
RX	21	1.706 (0.835-3.485)	0.143	-	-
R2	1	0.000 (0.000-Inf)	0.995	-	-
Histological grade	359	-	-	-	-
G1	55	Reference	-	-	-
G3	118	1.305 (0.826-2.063)	0.254	-	-
G2	175	1.156 (0.743-1.798)	0.521	-	-
G4	11	0.892 (0.311-2.560)	0.832	-	-

## Discussion

In this study, we systematically analyzed the expression profile and prognostic significance of different BSG transcript isoforms in liver cancer using data from TCGA and GTEx. Our results showed that ENST00000353555, encoding the basigin-2 isoform, is the dominant BSG transcript in both normal and tumor liver tissues. This finding is consistent with previous reports that basigin-2 is the most abundant isoform in various types of cancers [9,13,14]. Importantly, we found that ENST00000353555 expression was significantly upregulated in hepatocellular carcinoma (HCC) tissues compared to normal and tumor-adjacent liver tissues, suggesting a potential oncogenic role of basigin-2 in liver cancer.

Survival analyses further revealed that higher expression of ENST00000353555 was significantly associated with shorter OS and PFI in HCC patients. Notably, multivariate Cox regression analysis confirmed that ENST00000353555 expression was an independent prognostic factor for OS, after adjusting for clinicopathological factors such as AJCC stage. These findings are in line with previous studies reporting the unfavorable prognostic value of BSG overexpression in HCC [2,15,16]. However, our data provide novel insights by demonstrating that the basigin-2 isoform, encoded by ENST00000353555, is the key contributor to the poor prognosis associated with overall BSG expression in liver cancer.

The differential prognostic value of BSG transcript isoforms observed in this study may be attributed to their distinct biological functions and regulatory mechanisms. Previous studies have shown that basigin-2 (encoded by ENST00000353555) possesses stronger oncogenic properties compared to other isoforms, such as promoting tumor cell proliferation, invasion, and angiogenesis [9]. In contrast, the basigin-3 isoform (encoded by ENST00000545507) has been reported to inhibit the proliferation and invasion of HCC cells [10]. These functional differences may underlie the distinct prognostic significance of the BSG transcript isoforms in HCC.

The strong prognostic value of ENST00000353555 expression suggests that this transcript isoform could serve as a promising biomarker for risk stratification and prognostic prediction in HCC patients. Monitoring the expression levels of ENST00000353555 may help identify high-risk individuals who could benefit from more intensive treatment and surveillance strategies. Additionally, targeting the basigin-2 isoform or its associated signaling pathways could be a potential therapeutic approach for HCC.

It is worth noting that our study has some limitations. First, the analysis was based on retrospective data from TCGA and GTEx, and the findings need to be validated in independent patient cohorts. Second, the underlying molecular mechanisms by which the basigin-2 isoform contributes to HCC progression and poor prognosis remain to be elucidated. Future mechanistic studies are warranted to better understand the isoform-specific functions of BSG in liver cancer. Nevertheless, our findings provide important insights into the clinical implications of BSG transcript isoforms in HCC and highlight the potential utility of ENST00000353555 as a prognostic biomarker and therapeutic target.

## Conclusions

ENST00000353555 is an independent prognostic biomarker of unfavorable overall survival in patients with liver cancer. This finding suggests that the basigin-2 isoform encoded by ENST00000353555 may play a key role in the progression and poor prognosis of liver cancer. Monitoring the expression of this specific BSG transcript isoform could help identify high-risk individuals and provide more personalized treatment strategies. In addition, targeting the basigin-2 isoform or its associated signaling pathways may support a potential therapeutic approach for liver cancer patients. Additional mechanistic studies are necessary to elucidate the underlying molecular mechanisms by which ENST00000353555 contributes to the aggressiveness of liver cancer.
